# A Multilayer Ontology of Instruments for Neurological, Behavioral and Cognitive Assessments

**DOI:** 10.1007/s12021-014-9244-3

**Published:** 2014-09-21

**Authors:** Bénédicte Batrancourt, Michel Dojat, Bernard Gibaud, Gilles Kassel

**Affiliations:** 1 Inserm U 1127, CNRS UMR 7225, Sorbonne Universités, UPMC Univ Paris 06 UMR S 1127, Institut du Cerveau et de la Moelle épinière, ICM, F-75013 Paris, France; 2 GIN, U 836, INSERM, Université Joseph Fourier, Grenoble, France; 3 LTSI, U 1099, INSERM, Université de Rennes 1, Rennes, France; 4 MIS, EA 4290, Université de Picardie Jules Verne, Amiens, France; 5 Hôpital Pitié-Salpêtrière, 47 Boulevard de l’Hôpital, 75651 Paris Cedex 13, France

**Keywords:** Biomedical ontology, Scale, Neuropsychological dataset, Federated architecture, Mediation, Data integration, Neuroscience

## Abstract

Advances in neuroscience are underpinned by large, multicenter studies and a mass of heterogeneous datasets. When investigating the relationships between brain anatomy and brain functions under normal and pathological conditions, measurements obtained from a broad range of brain imaging techniques are correlated with the information on each subject’s neurologic states, cognitive assessments and behavioral scores derived from questionnaires and tests. The development of ontologies in neuroscience appears to be a valuable way of gathering and handling properly these heterogeneous data – particularly through the use of federated architectures. We recently proposed a multilayer ontology for sharing brain images and regions of interest in neuroimaging. Here, we report on an extension of this ontology to the representation of instruments used to assess brain and cognitive functions and behavior in humans. This extension consists of a ‘core’ ontology that accounts for the properties shared by all instruments supplemented by ‘domain’ ontologies that conceptualize standard instruments. We also specify how this core ontology has been refined to build domain ontologies dedicated to widely used instruments and how various scores used in the neurosciences are represented. Lastly, we discuss our design choices, the ontology’s limitations and planned extensions aimed at querying and reasoning across distributed data sources.

## Introduction

In neurosciences, imaging plays a central role providing information about brain structure and function. In particular, magnetic resonance imaging (MRI) generates anatomical and functional information on the healthy or diseased brain and is a cornerstone of cognitive neuroscience (Logothetis 2008; Raichle 2009).

### The Need for Ontologies of Instruments

To further investigate the complexity of the human brain, recent studies of large population cohorts have sought to cross-relate MRI markers with biomarker levels, cognitive parameters and behavioral scores. The studies’ main objectives are to (i) relate aspects of brain morphology to human behavior and cognitive performance levels and (ii) investigate the underlying neural mechanisms. The NeuroLOG project^1^ was launched with the objective of facilitating the sharing of neuroimaging data and image-processing resources via an ontology-based, federated approach (Gibaud et al. 2011). OntoNeuroLOG was developed during the four-year NeuroLOG project (2007–2010) and was used to link four French imaging repositories: Paris Pitié-Salpêtrière, Grenoble Institute for Neurosciences, INRIA Sophia-Antipolis and VISAGES. This ontology was designed with the concrete goal of sharing instrument-based assessment results in the context of the NeuroLOG project and NeuroLOG platform. However, the overall ontology and the core ontology of instruments were designed to help model a much wider range of instruments than is required in the NeuroLOG project. The project shares a number of features with the Biomedical Informatics Research Network^2^ initiative, which pioneered work on federated data integration and provided proof of concept of the application of ontology-based mediation to neurosciences research (Martone et al. 2004). Computer scientists, biologists (Hill et al. 2010) and neuroscientists (Van Horn and Toga 2009) now broadly agree that ontology development is an essential issue when capturing, storing, representing and then sharing knowledge about a specific biomedical domain. In particular, the NeuroLOG project proposed a multilayer application ontology (OntoNeuroLOG) for the specification of common semantics when sharing brain images (Temal et al. 2008). In the work presented here, we provided OntoNeuroLOG with an ontology of instruments used to assess brain and cognitive (dys) functions, behaviors and psychological states in humans.

### How Our Work Is Positioned

There are several international efforts addressing the representation of “cognitive neurosciences” information. For our purposes, to model instruments we need to combine abstract and concrete terms. Indeed, if we consider the NCBO BioPortal (today’s largest resource for biomedical ontologies) several ontologies were relevant: from the 369 published ontologies, 104 concern methods and tools for evaluating brain functions (retrieved using six keywords: Assessment, Instrument, Score, Scale, Test and Questionnaire) and 148 refer to brain functions (retrieved using 19 keywords: generic keywords such as Cognition, Emotion or Sensation; domain keywords such as Working Memory, Episodic Memory or Executive function; or quality and measure keywords such as Intelligence quotient, Verbal fluency or Perseveration, and instrument or test keywords such as Rey Figure, STROOP or CDR scale).

We analyzed the nine most relevant ontologies (RCD, NCIT, SNOMED, NIFSTD, BIRNLex, COGAT, LOINC, SYN and ERO), in order to determine how pivotal concepts such as ‘Instrument’ and ‘Assessment’ were modeled. We concluded that these ontologies do not describe the internal structure of instruments because the latter are considered as physical objects (generally linked to a ‘Device’ (BFO) or a ‘Medical Device’ (UMLS)). The NIFSTD ontology (Version 2.9.6.1 of September 3, 2013) adopts the most interesting approach, in which a classification of “Assessments” (inherited from BIRNLex) is provided as a subclass of “Protocol application” which in turn is a subclass of “Planned process” (and is thus related to the realization of a “Plan”). However the part addressing assessment instruments is neither complete nor fully consistent. Instruments are assessments without explicit models. Moreover, the notion of sub-instrument is not present and then no explicit relation exists between the global score provided and the underlying sub-instruments scores that compose it. Variables measured by an instrument are not introduced and finally there is no explicit distinction between the tool for investigation (i.e. instrument), the investigation process (i.e. instrument assessment) and the score obtained. These models represent neither the internal structure of instruments nor the resulting scores, assessment actions and variables used to refer to the qualities measured. We strongly consider that models with these characteristics are required and should be managed consistently across the broad range of existing instruments. Our aim with our instrument ontology was to formally represent all these concepts.

### Our Ontological Approach

Our prime objective is to facilitate the sharing of cognitive and behavioral scores in the federated systems that underpin multicenter research studies and clinical trials in the neurosciences. As with the ADNI and HCP initiatives, the intention is to correlate cognitive and behavioral scores with imaging markers and biomarkers.

Many different “assessment instruments” are used in the neurosciences. This diversity is driven by the need to capture the many facets of human brain function and behavior and the very broad spectrum of symptoms associated with brain dysfunction. It also results from clinicians’ and psychologists’ on-going efforts to improve existing instruments and introduce new ones, in order to assess brain functions in ever greater detail (White and Hauan 2002).

To build our ontology of instruments, we adopted a multilayer, multicomponent approach that had already been implemented within the NeuroLOG project (Temal et al. 2006). In fact, OntoNeuroLOG is organized into sub-ontologies (modules) situated at three different levels of abstraction. At the most abstract level, the DOLCE foundational ontology (Masolo et al. 2003) provides a set of abstract concepts (e.g. *physical object* and *quality*) and relations (e.g. *part-whole*, *constitution*, etc.) for structuring any kind of domain by specialization. DOLCE is supplemented here by a few formal ontologies, such as a formal ontology of artifacts (Kassel 2010). At an intermediate level, “core” domain ontologies (Gangemi and Borgo 2004) define a minimal set of generic and key concepts (e.g. *subject*, *domain* and *score*) for each domain concerned. Lastly, at the most specific level, core domain ontologies are in their turn refined via the introduction of specialized, domain-specific concepts (e.g. *stroke*, *evoked potential* and *thrombolysis* in neurology). This multilevel abstraction approach consists in applying the same set of generic principles to the conceptualization of domains covered by application ontologies. The main objective here is to facilitate the development and maintenance of ontologies and to ensure a high degree of cross-domain consistency (Smith and Scheuermann 2011).

The ontology presented here includes modules situated both at the intermediate level of core domain ontologies and at the most specific level of domain ontologies. Our core domain ontology (presented in part at the Formal Ontology in Information Systems conference (Batrancourt et al. 2010)) seeks to capture the essential neuropsychological and psychometric properties of a number of instruments, including: (i) their decomposition into sub-instruments, (ii) the definition of associated variables (leading to scores) and (iii) the current domains and qualities explored and measured by these instruments and variables. The core domain ontology was supplemented with domain ontologies, each of which conceptualizes specific kinds of instrument (e.g. the Wechsler Adult Intelligence Scale (WAIS) and the Expanded Disability Status Scale (EDSS)). In this case, an instance represents an instrument administered at a particular center (e.g. *EDSS – Pitié-Salpêtrière Hospital (Paris, France)*).

The ontology is available at the BioPortal repository (https://bioportal.bioontology.org/ontologies/ONL-MSA). Its implementation (specification and use) as a component of a specific federated architecture for facilitating data sharing in distributed data centers has been described in (Michel et al. 2010) and (Gibaud et al. 2011). In this paper we focus on the contents of the ontology.

The remainder of this paper is organized as follows. In Section 2, we describe the generic modules of OntoNeuroLOG that were reused for our present work. In Section 3, we describe our core ontology of the domain of instruments; it covers instruments, actions corresponding to the administration of instruments and the scores obtained as a result. In Section 4, we present specializations of the core ontology (domain ontologies) modeling three specific instruments that are widely used in clinical practice^3^: the Mini-Mental State (MMS) or Mini-Mental Status Examination (MMSE), the Expanded Disability Status Scale (EDSS) and the Clinical Dementia Rating (CDR). Lastly, we discuss our design choices, the current limitations of our conceptualization and planned extensions in Section 5.

## Our Ontological Reference Framework

**Fig. 1:**
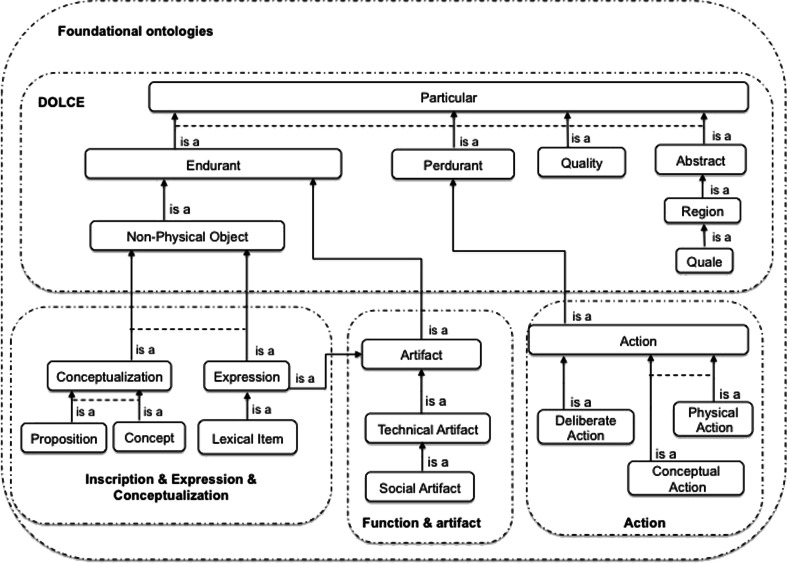
An excerpt of our taxonomy of concepts at the foundational level. A dashed rectangle delimits a specific ontological module; a solid arrow represents a subsumption link (i.e. an “is a” relation); a dashed line indicates that sibling concepts are incompatible (i.e. they have disjoint extensions)

Here, we provide a brief reminder of the main structuring principles and concepts that underlie important modules reused in the present work. An excerpt of our foundational concepts’ taxonomy is shown in Fig. 1.

### Particulars (DOLCE)

The DOLCE ontology (C. Masolo et al. 2003) constitutes the keystone of OntoNeuroLOG. DOLCE’s domain is that of Particulars,^4^ that is to say entities that cannot be instantiated (e.g. “my car”) rather than *universals* (e.g. “being a car”).

Four sub-domains of Particulars are distinguished (see Fig. 1):

- Endurants^5^ are entities “enduring in time”, which are *primarily* directly related to space. Physical objects (e.g. a pen, a printed copy of an article) are typical Endurants. Besides Physical objects, DOLCE considers a class of Non-physical Objects. The distinction between Physical Objects and Non-physical Objects corresponds to the difference between two realities or *modes* of existence. Basically, Non-Physical Objects exist insofar as agents conventionally create them and speak about them. The domain of Non-Physical Objects covers entities whose existence depends on either an individual (Mental Objects, e.g. a private mnemonic method, or the content of this sentence that you interpret) or a community of agents (Social Objects, e.g. a company, the stipulations of a law).
- Perdurants are entities “occurring in time”, which are *primarily* directly related to time. Perdurants are generated by Endurants: the latter temporarily participate in (participatesInDuring) the former.
- Endurants and Perdurants have Qualities that we perceive and/or measure (e.g. the weight of a printed copy of this article and the time spent reading this article). Note that Qualities are inherent to the entity that bears them, since they are characteristic of their bearer and present throughout its existence.
- Qualities temporarily occupy positions within Regions. Some Regions called Qualia (Quale in the singular) are defined as atomic Regions (e.g. “25 g in weight” and “20 min in duration”). Other Regions are *mereological* sums (sums of parts) of Qualia. For instance, the Region of colors named ‘red’ may be considered as having for parts the Qualia named “Scarlet” and “Crimson”. The sum of all Qualia associated with a Quality kind is called a (Quality) Space. Spaces in DOLCE are similar to Gärdenfors’ *conceptual spaces* (Gärdenfors, 2000).

As is commonly the case, the development of ontologies in a particular domain requires the prior extension of the foundational resources. For example, in order to conceptualize the assessment of a subject’s ability to walk a certain distance, we need to have the generic notions of *ability* and *action* at our disposal (and these are not present in DOLCE). Similarly, to model the act of taking a patient’s temperature with a thermometer, we need the generic notions of *instrument* and *measurement instrument*. In the remainder of this section, we complete DOLCE with some generic modules needed to conceptualize our application domain.

### Inscriptions, Expressions, and Conceptualizations

In the present work, we consider intangible (i.e. non-physical) instruments as contents of documents specifying rules for measuring the subject’s state, behavior or brain function. These instruments contrast with physical instruments (e.g. thermometers and computed tomography (CT) scanners). To model these conceptual contents, we reused the Inscription & Expression & Conceptualization (IEC) module (Fortier and Kassel 2004), which provides a set of basic contents for handling the generic notion of *document*. The IEC module is a core ontology in the domain of information and information-bearing entities. It extends DOLCE by introducing three main kinds of entities:

- Inscriptions (e.g. printed texts and computer files) are physical knowledge forms materialized by a substance (e.g. ink or an electrical field) and inscribed on a physical support (e.g. a sheet of paper or a hard disk). In addition to their materiality, one important characteristic of Inscriptions lies in their “intentional” nature (meaning that these entities count as other entities). For example, Inscriptions count as Expressions.
- Expressions (e.g. texts and logical formulae) are non-physical knowledge forms ordered by a communication language. Expressions are physicallyRealizedBy Inscriptions and, like Inscriptions, they are intentional entities conveying contents for agents.
- Conceptualizations consist of the ultimate means by which agents can reason about a world. Two kinds of Conceptualizations are distinguished: Propositions, as a means of describing states of affairs, and Concepts, as a means of classifying entities. Note that, as for the practical semiotics^6^ introduced in the SUMO ontology (Pease and Niles 2002), Propositions may encompass the content expressed by sentences, theories, books and even libraries.

We shall see in Section 3 that Propositions correspond to the intrinsic nature of our assessment instruments. Meanwhile, we extend DOLCE in another way, in order to account for *actions*.

### Actions and Participation Roles

The instruments are administered in order to evaluate the subjects’ state, behavior or brain function. The administration of these instruments and the assessment of the subjects’ state are actions - in other words, events intentionally carried out by agents. The design of an instrument is itself an action. To account for this notion of *action*, we use a minimum set of concepts (see Fig. 1):

- Actions are Perdurants controlled by an intention. They contrast with Happenings, which lack an intentional cause.
- Deliberate Actions are premeditated actions. According to current philosophical theories of actions, Deliberate Actions are controlled by a *prior* intention that consists in planning the action (before its initiation) and then in controlling it in a rational way (Pacherie 2000).
- According to another classification dimension, Physical Actions (whose effects bear on Physical Endurants, e.g. *curing a patient*) are distinct from Conceptual Actions (whose effects bear on Conceptualizations, e.g. *acquiring data from subjects*).

Various entities participate in these Actions in different ways, in the sense that they have different roles. As a complement to DOLCE, our “Participation role” module specializes the participation relation participatesInDuring to account for specific ways in which Endurants participate temporally in Actions (e.g. isAgentOfAt, isInstrumentOfAt, isResultOfAt). In turn, these relations are used to define *participation roles* specializing the concept Endurant (e.g. Agent, Instrument or Result). However, participation roles do not define the essence of the entities playing these roles. For example, an entity playing the role of an Agent needs to possess a disposition (or capacity) to intentionally control events. This type of disposition may be owned by various entities: a human being, an organization, a robot or a sophisticated sensor. Entities playing the role of an Instrument are generally technical artifacts that have been intentionally produced for that very purpose. These artifacts may be physical sensors or conceptual measurement procedures. In the next section, we introduce a set of concepts to reflect the essence of the artifacts.

### Artifacts

Artifacts are commonly defined as “*entities intentionally made or produced for some reason*” (Hilpinen 2004). To be able to describe the two main dimensions characterizing these entities (namely *being intentionally produced* and *being produced for a certain reason*), we reuse concepts from our ontological module “Function &Artifact” (Kassel 2010). According to this ontology:

- Artifacts are the result of an intentional production and thus have an Author.
- Artifacts are produced for a certain reason. Various kinds of reasons (and hence various types of Artifacts) are considered: to convey an emotion and be of aesthetic interest (for works of art) or enable their author (or another agent) to do something (for “functional” or Technical Artifacts). The latter are Artifacts to which a Function is ascribed, given that a Function is defined as an “*acknowledged capacity to enable the realization of a kind of action*” (Kassel 2010).
- Within Technical Artifacts, Private Artifacts are distinguished from Social Artifacts according to whether the function in question is ascribed by an individual or a community of agents.

It is important to note that DOLCE’s distinction between Physical and Non-Physical Objects transcends the domain of Artifacts. Indeed, the latter are defined by the *origin* of their existence (i.e. their intentional production) rather than a *mode* of existence (i.e. their dependence vis-à-vis agents who conventionally create, make use of and communicate about them (C. Masolo et al. 2004)). This difference explains why we are able to distinguish between physical artifacts (e.g. a CT scanner) and non-physical artifacts (e.g. assessment rules expressed in a document). To account for this distinction, we consider that Technical Artifacts (i) possess an internal (physical, social or cognitive) essence, (ii) have been intentionally produced and (iii) necessarily have a Function.

## A Core Ontology of Assessment Instruments

As emphasized above, a wide range of assessment instruments exists. Some are very simple, with just a few indicators (“indicator” is the term used in (Bilder et al. 2009)), whereas others involve a great number of indicators. Some assessment instruments are limited to recording a subject’s answers to a questionnaire concerning his/her behavior or psychological state. Others involve several tests, each of which is characterized by several parameters quantifying the subject’s performance. The way indicators are coded also varies greatly from one instrument to another. Some instruments refer to a predefined scale with discrete qualitative or quantitative values, whereas others have a continuous value within an open or closed interval. Moreover, some instruments produce directly meaningful results, whereas others require further processing: for instance, the subject’s age, level of skill or educational level may be required for correct interpretation of the instrument’s output. In order to address this complexity, we sought to identify the common features of a broad range of instruments. We thus designed a core model of instruments that highlights the common structure of instruments, their function (i.e. the kind of measured quality and the domain explored) and how the result of their assessment is recorded, in order to provide a taxonomy of instruments.

In terms of common features, we are concerned with situations in which health professionals administer instruments (tests and questionnaires) to assess a subject’s behavior and cognitive performance. To model these situations, we consider two main entities: the actions carried out (so-called “instrument-based assessments”) and the instruments used. In Section 3.1, we detail the part of the core ontology that conceptualizes these two entities. Instruments are structurally and functionally complex entities. They are composed of items (named “instrument variables”) that measure specific aspects of the subject’s neurologic state, behavior or cognitive performance in the domain explored by the instrument. The administration of instruments is thus further decomposed into variable assessments, which consist in asking questions or requesting tasks to be completed. These assessments yield in scores, which are derived from the subject’s responses or behavior. In Section 3.2, we add to our presentation of the core ontology by conceptualizing “instrument variables” and “variable assessments’.

### Instrument-Based Assessment

Instrument-Based Assessment actions consist in acquiring data from Subjects by administering an instrument.

**Fig. 2:**
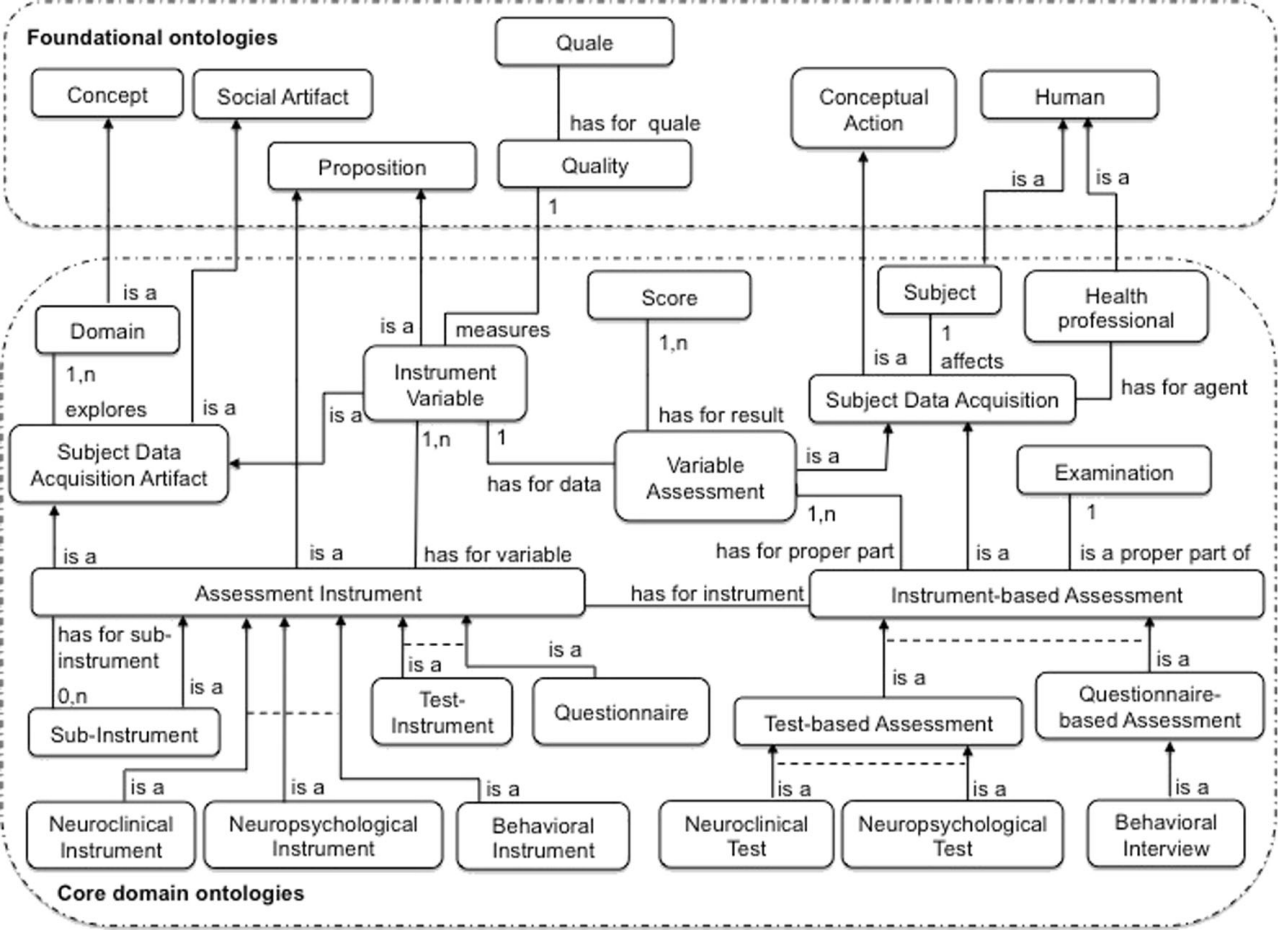
Concepts and relations structuring one part of our core ontology of instruments. A solid arrow represents a subsumption link (i.e. an “is a” relation); a dashed line indicates that sibling concepts are incompatible (i.e. they have disjoint extensions)

The conceptualization of these actions has a pivotal role in our ontology by connecting a large number of entities (see Fig. 2): a Health Professional (for instance a Neuropsychologist or a Neurologist) involved as an Agent, the Subject (either a Healthy Volunteer or a Patient) involved in data acquisition, the broader context of the data acquisition (i.e. an Examination within a Study), the instrument used (which prescribes the data to be acquired and the way they are acquired) and, lastly, the scores generated by the questions and/or tests administered.

There are generally two main forms of Assessments. The first are called Test-Based Assessments and solicit an authentic production from the Subject, e.g. a reflex, a performance (such as drawing or a 500-m walk). The second are called Questionnaire-Based Assessments and consist of an interview or an inventory. In all cases, these are complex actions whose structure is based on that of the instrument administered. At the finest level of decomposition, one finds *items* that prompt the performance of measurements; these actions and their results are detailed in Section 3.2. In our ontology, we adopt a common classification of Instrument-based Assessments that depends on the type of acquired data (e.g. neuropsychological/cognitive, behavioral or neuroclinical/neurologic data). However, this classification does not induce formal rules about who is allowed or not to administering them.^7^

Instrument-Based Assessments are organized around the administration of an “Assessment Instrument” (an “Instrument”, for short). These Instruments are intentionally designed to assess the subject’s state under one or more dimensions. According to our theory of artifacts (cf. Section 2.4), Instruments clearly are Technical Artifacts that may be described in three respects. Instruments are:

- Intangible, i.e. propositional contents, including “clearly defined methods and instructions for administration or responding, a standard format for data collection, and well-documented methods for scoring, analysis, and interpretation of results” [CDISC, 2008].^8^
- Functional, i.e. tools enabling to explore entities related to the Subject’s state. These categories of entities correspond to the Instrument's Domain(s).
- Social, i.e. intentionally created, adopted for use, adapted and maintained by a community that ascribes them with the status of a standard.

As intangible, propositional content, an Instrument is expressed in language and is physically inscribed on a medium of some kind. Indeed, Instruments are usually physically materialized by several documents.^9^ In order to conceptualize Instruments, we chose to focus on their conceptual structure and function.

Structurally, an Instrument appears as a list of items, each of which corresponds to a specific aspect of the subject’s state. Questions or tasks are associated with each item assessing the subject’s performance levels. We refer to these parts as “Instrument Variables”, in order to emphasize that they are measurement tools and that they bear values (which vary from one subject to another). But some Instruments have a more complex structure. Given that (i) brain function is constituted by a number of smaller elementary processes and (ii) the most complex^10^ functions are made up of a large number of elementary functions distributed across many areas of the brain, some Instruments are composed of Sub-Instruments. This is notably the case for the MMS Instrument, which notably contains MMS Orientation and MMS Language tests. Each Sub-Instrument explores a more elementary process of overall cognitive function (e.g. orientation, memory, verbal capacities, etc.).To model the structure as a whole, we use the isAPartOfDuring relation (or, more precisely, the isAProperPartOfDuring sub-relation).

In functional terms, Instruments explore classes of entities with differing ontological natures. For instance, memory is a cognitive capacity or function, whereas depression is a disease state. Given that the effects of brain disorders are rarely confined to a single behavioral dimension or functional system (Lezak et al. 2004) (pp. 86–87), Instrument-Based Assessments focus on different issues: neurologic disorders (e.g. weakness, stiffness and visual impairments), cognitive impairments (e.g. aphasia, failure of judgment and lapse of memory) and other behavioral disorders (e.g. personality change, reduced mental efficiency and depression). To measure these various features, Instruments are specialized: Questionnaires mainly explore behaviors and disease states (called “traits”) while Tests (called “Test Instruments” so as not to confuse them with the act of testing a subject) mainly explore abilities, skills, cognitive impairments and unaffected cognitive functions. The structural complexity of an instrument is related to its functional complexity. An Instrument may be designed to explore one or more domains (Mono-domain *vs.* Multi-domain Instrument). The WAIS-III is a typical example of a Multi-domain Instrument; it explores a whole set of domains, like “verbal comprehension”, “working memory”, “perceptual organization” and “processing speed”. This reflects Wechsler’s definition of intelligence as “the aggregate or global capacity of the individual to act purposefully, to think rationally and to deal effectively with his environment” (Wechsler 1939).

Formally, a Domain is modeled as an individual concept that classifies classes of entities. The concepts of states, capacities and disease states (having a role as an Instrument’s Domain) are reified^11^ as individuals in the domain of discourse. This means that a concept such as Verbal comprehension (which in principle represents a class of dispositions of subjects) is accounted for in our conceptualization as an individual (an instance of the class Concept). This enables to assign it with properties and, for instance, express the fact that the Verbal comprehension domain is explored by Instruments in the WAIS-III class.

In this section, we showed how instruments and the administration of instruments were conceptualized. The following section focuses on the Instrument Variables and the latter’s two main functions: (i) to describe precisely what is being explored and measured and (ii) to relate the scores to the context in which the assessment is made.

### Variable Assessment

**Fig. 3:**
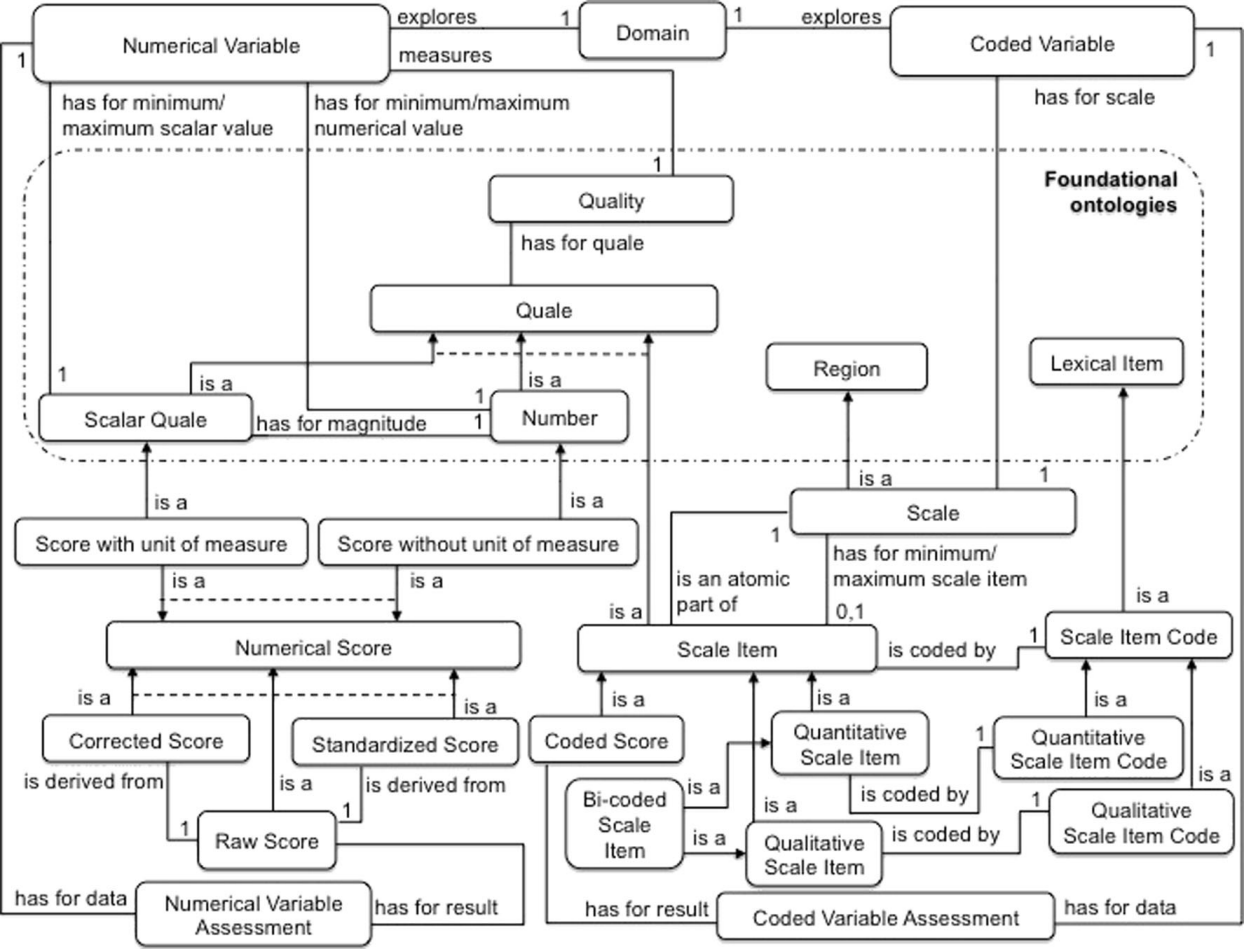
Concepts and relations supplementing our core ontology of instruments. A solid arrow represents a subsumption link (i.e. an “is a” relation); a dashed line indicates that sibling concepts are incompatible (i.e. they have disjoint extensions)

An Instrument is composed ultimately of Instrument Variables (“Variables”, for short) that prescribe a specific measure. As with the conceptualization of Instruments, we consider two main entities: Variables and the measuring actions associated with them (called “Variable Assessments”) (Fig. 3).

Variables are Artifacts (Subject Data Acquisition Artifacts, in fact) with specific functions. As for Instruments at the most general level, our conceptualization neglects the documentation associated with Variables (e.g. the questions that have to be asked and instructions on how to execute tests) and focuses on functional descriptions. In functional terms, a Variable explores a Domain that usually corresponds to or refines the Domain explored by the corresponding instrument. Depending on whether this Domain coincides with the Instrument’s Domain or it relates to other similar entities (with the aim of acquiring additional information), one distinguishes between a Main Variable and a Secondary Variable, respectively. Furthermore, a Variable aims at measuring a dimension or property of these Domain entities, which we conceptualize as a Quality (for example, the Intensity, the Frequency, the Severity or the Impact of the patient’s disease state on his/her relatives). The explored Domain and the measured Quality constitute the functional information attached to a Variable.

A Variable Assessment action isAProperPartOf an Instrument-based Assessment action. These actions share several properties: they have the same Agent and concern the same Subject. The Variable Assessment actions link scores (as results of measurements) to the measured Variables. Depending on the nature of the explored Domain, Qualities may have as value i) a Number or a Scalar Quale (a Number plus a Unit of Measure) – the corresponding Variable is called a “Numerical Variable”, or ii) coded items of a Scale – the corresponding Variable is called “Coded Variable” (see Fig. 3). Numerical Variables essentially measure the level of performance in the realization of actions. The measured values are thus (for example) elapsed times, distances covered and numbers of items recognized. A Numerical Variable is often associated with intervals of allowed values, which we conceptualize as minimum and maximum values. For example, the Vocabulary Variable of the WAIS-III Vocabulary instrument measures the subject’s verbal knowledge and understanding on a scale of 0 to 66. For Coded Variables, the measured values are items of a scale (Scale Items) encoded by linguistic expressions (to facilitate the communication of values between humans) and/or numbers (to enable calculations). For Coded Variables, the measured values are items of a scale (Scale Items) encoded by linguistic expressions (to facilitate the communication of values between humans) and/or numbers (to enable calculations). For example, the EDSSv1 Variable of the *Expanded Disability Status Scale* (EDSS) neurologic instrument measures the subject’s neurologic state on a scale that hasForMinimumScaleItem: “0.0: Normal neurologic exam” and hasForMaximumScaleItem: “10.0: Death due to MS”. It is important to note that for a given Quality, a great number of Subjects share the same value as a measurement result.^12^ The Score concept’s role is therefore to specify which value is the Result of a Variable Assessment by linking a given Variable to a given Subject. Some scores are sensitive to age, gender or educational level. Thus, for many Instruments based on population screening, normative data has been published as function of age, gender and/or educational level. This information is included in the definition of the Instrument. For example, WAIS-III included normative data for the *IQ* Variable separated into 13 age-dependent groups. Variables are then categorized as Gender-Dependent Variables, Age-Dependent Variables and Cultural-Skill-Dependent Variables. Normative data are often used to quote individual Scores obtained by Subjects. In such a case, Raw Scores are converted to Corrected Scores and Standard Scores by using charts and tables provided with the Instrument.

## Domain Ontologies for Three Specific Instruments: The MMS, EDSS and CDR

The objective of this section is to illustrate the use of the above-described core ontology to define specific ontologies of instruments through concept specialization. In the previous sections, we described our generic conceptualization of instruments and highlighted the latter’s common structure. In the present section, we show how this core ontology is used to model the MMS (which grades cognitive functions with numerical variables), the EDSS and the CDR (both of which express neurologic and behavioral assessments as coded variables), We introduce the instruments’ respective general structures and decompose them into sub-instruments and variables. The specific instruments and instrument variables are defined as subclasses of the generic classes of Instruments and Instrument variables. These classes model the common properties of the corresponding instrument and variable instances used at different healthcare institutions. Alternatively, we could have chosen to model these entities as instances and not as classes. However, our ontology was primarily intended for use as a common reference ontology, in order to integrate score data from different neuroimaging centers and obtained from assessment instruments that may slightly differ from one site to another. Modeling the common properties of these instruments as classes is appropriate and provides some flexibility in the definition of local instrument instances.

### The Mini-Mental State as an Example of a Neuropsychological Instrument

A neuropsychological check-up is based on the observation and application of objective Tests for grading cognitive functions. A neuropsychological examination is structured by the list of the cognitive functions to be tested, e.g. executive function, memory, language, attention, arithmetic, logical reasoning, global cognitive efficiency, movements and visuospatial functions – each of which is explored by one or several Neuropsychological Instruments. Some of the latter may be composed of several Sub-Instruments, each of which is specifically designed to explore a particular cognitive function.

The examination performed by a Neuropsychologist as an Agent generates both qualitative and quantitative Scores. The MMS is probably the most widely used neuropsychological instrument in dementia assessment (Lezak et al. 2004) (pp. 706–708) and is used routinely to grade cognitive functions: “we devised a simplified, scored form of the cognitive mental status examination, which includes eleven questions, requires only 5–10 min to administer, and is therefore practical to use serially and routinely. It is “mini” because it concentrates only on the cognitive aspects of mental functions, and excludes questions concerning mood, abnormal mental experiences and the form of thinking” (Folstein et al. 1975). The MMS mainly assesses verbal functions, memory abilities and construction. The MMS score decreases with age and increases in proportion to the subject’s educational level.

**Fig. 4:**
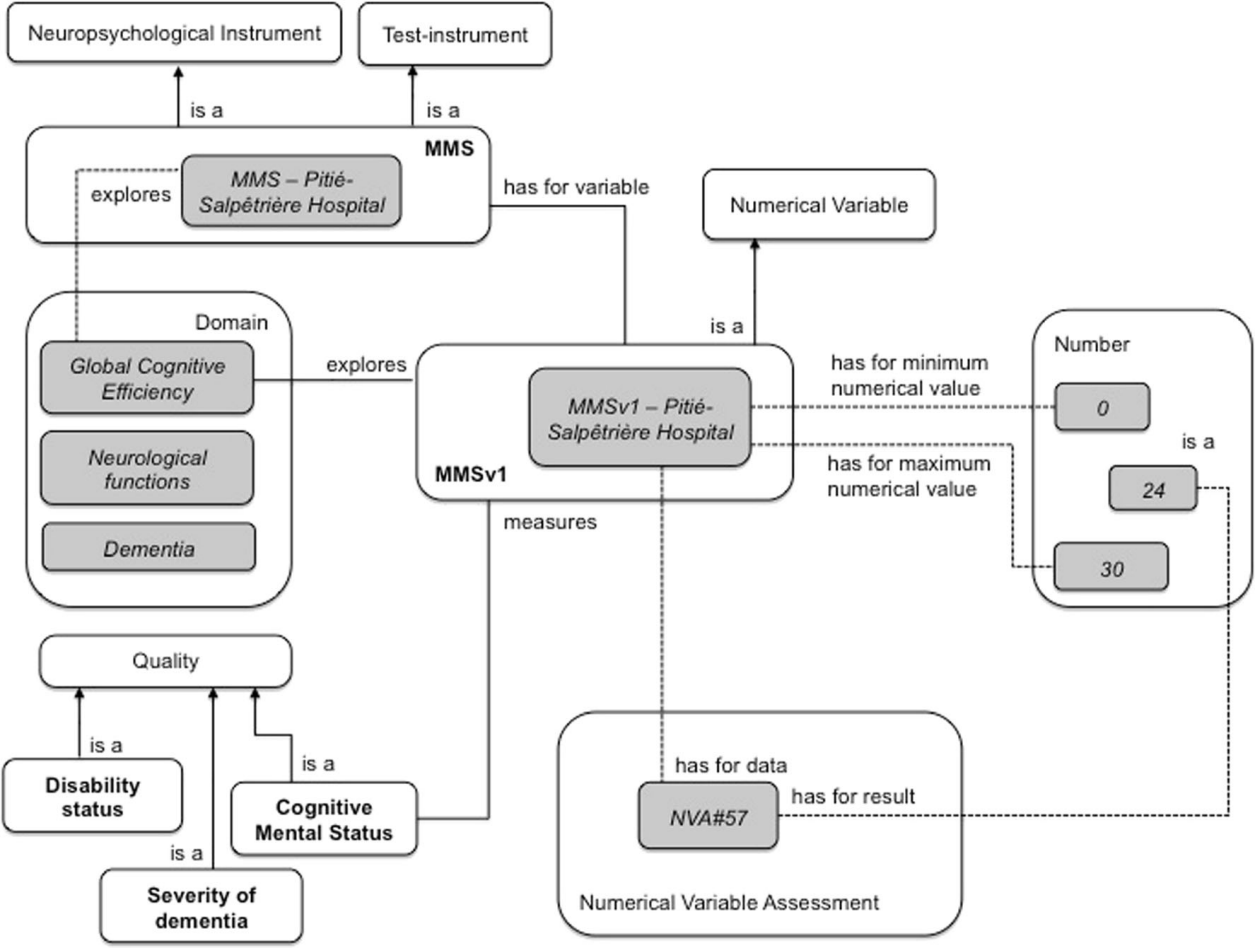
The main concepts and relations used to represent the MMS instrument. The instance MMS-Pitié-Salpêtrière Hospital represents a specific MMS instrument used at Pitié-Salpêtrière Hospital (Paris, France). The Figure illustrates the MMSv1 variable used for rating, based on various Numerical Scores for cognitive mental status at Pitié-Salpêtrière Hospital. The *NVA#57* hasForResult a Numerical Score equal to 24, in this case. White rectangles represent domain ontology concepts; gray rectangles represent instances

Hence, MMS is a Neuropsychological Instrument that explores the domain of GlobalCognitiveEfficiency (see Fig. 4). It is composed of five Sub-Instruments and several Variables (see Table 1) for assessing five domains: orientation, calculation, language, memory and praxis. The MMS and its Sub-Instruments are Test Instruments because they solicit an authentic production from the subject (e.g. drawing, writing and word retrieval).

**Table 1:** The neuropsychological instrument *Mini-Mental State Examination (MMS)* with its sub-instruments and instrument variables

Instrument acronym	Variable acronym	Domain explored by variable	Maximum numerical value
Instrument name		Quality measured by variable	
MMS	MMSv1	Global cognitive efficiency	30
Mini Mental State		Cognitive mental status	
MMS-1	MMS-1v1	Orientation	10
MMS Orientation		Performance on orientation	
MMS-1-1	MMS-1-1v1	Orientation to time	5
MMS Orientation to time		Performance on orientation to time	
MMS-1-2	MMS-1-2v1	Orientation to place	5
MMS Orientation to place		Performance on orientation to place	
MMS-2	MMS-2v1	Short term verbal memory	3
MMS Registration		Performance on registration of three objects	
MMS-3	MMS-3v1	Attention	5
MMS Attention and Calculation		Performance on counting backwards by 7	
MMS-4	MMS-4v1	Long term verbal memory	3
MMS Recall		Performance on recall of three objects	
MMS-5	MMS-5v1	Language	8
MMS Language tests		Language performance	
MMS-5-1	MMS-5-1v1	Oral language production	2
MMS Language naming		Performance on naming of two objects	
MMS-5-2	MMS-5-2v1	Oral language production	1
MMS Language repetition		Performance on repetition of a sentence	
MMS-5-3	MMS-5-3v1	Oral language comprehension	3
MMS Language 3 stage command		Performance on execution of a 3 stage command	
MMS-5-4	MMS-5-4v1	Written language comprehension	1
MMS Language reading		Performance on reading a sentence	
MMS-5-5	MMS-5-5v1	Written language production	1
MMS Language writing		Performance on writing a sentence	
MMS-6	MMS-6v1	Motor component of constructional functions	1
MMS Copy design		Copy accuracy	

All of the MMS’s Variables are Numerical Variables (see Fig. 4). For example, the variable MMSv1 (range: 0 to 30) is a Numerical Variable, which measures the quality Cognitive Mental Status and hasForQuale a number (an integer) between 0 (MMSv1 hasForMinimumNumericalValue 0) and 30 (MMSv1 hasForMaximumNumericalValue 30). MMSv1 isADataOf a Numerical Variable Assessment which hasForResult a Numerical Score (a subclass of Score). The MMS provides dimensionless Scores. Lastly, the Numerical Variable Assessment of the variable MMSv1 hasForResult a Score that sums the scores of all the Numerical Variable Assessments of the MMS's Sub-Instruments.

### The Expanded Disability Status Scale as an Example of a Neurological Instrument

The EDSS measures disability, neurologic dysfunction and disease severity in multiple sclerosis (Kurtzke 1983). It consists of a neurologic evaluation where walking and motor control ability contribute mainly to the EDSS final score, in addition to brainstem, sensory, bowel, bladder, and visual capacity examination (Lezak et al. 2004), (pp. 244–245).

A Neurologist administers EDSS to explore neurologic functions in multiple sclerosis patients and thus to estimate the disease severity. This Instrument is representative of the category of Neurologic Instruments, used to rate the degree of difficulty encountered by the subject in performing a task that involves a disease-altered brain function. The EDSS is a Test-Instrument (rather than a Questionnaire), since it primarily relies on the subject’s actual performance (e.g. the presence of reflexes and the subject’s performance in a 500-m walk (see Fig. 2)).

**Fig. 5:**
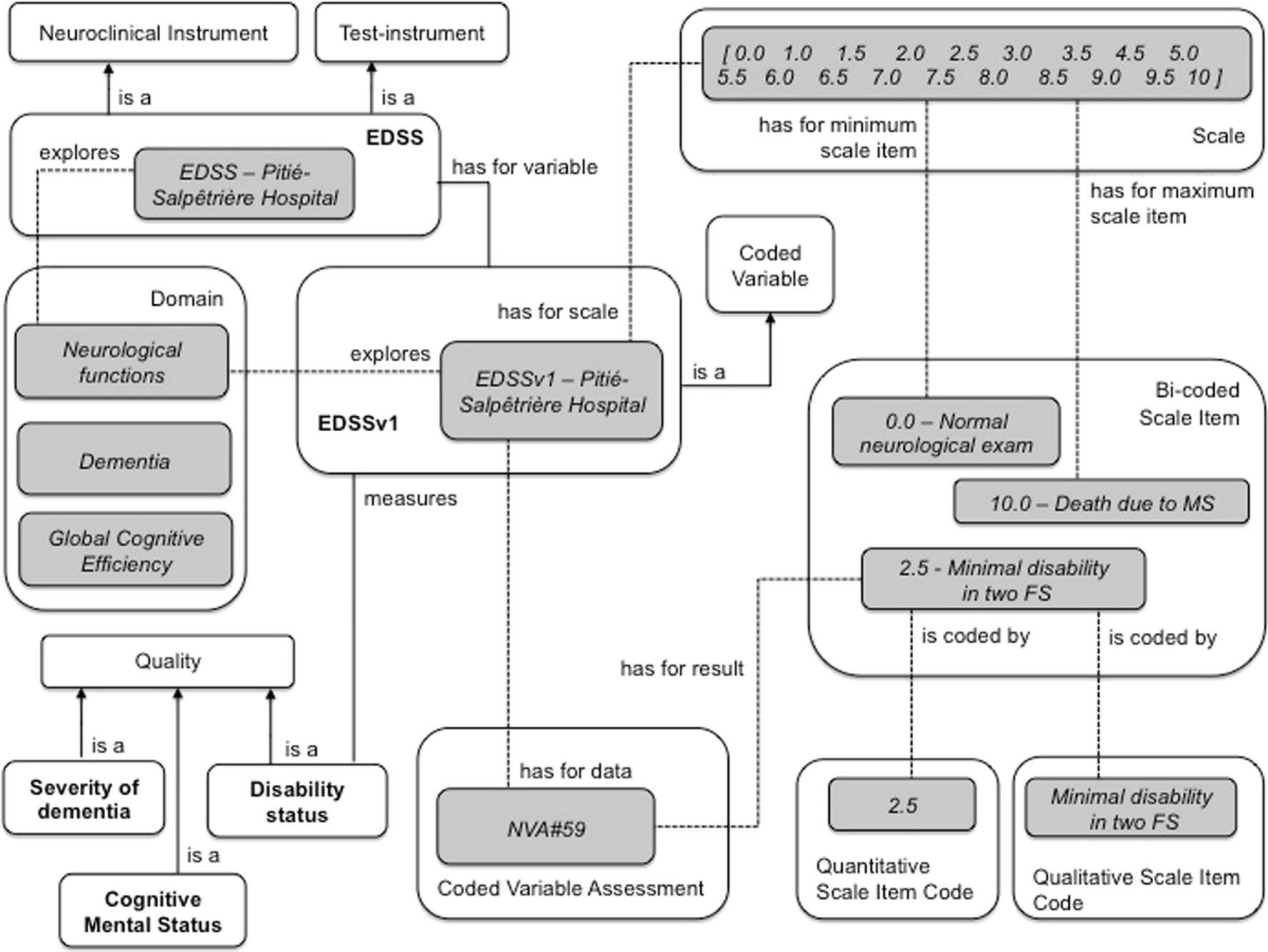
The main concepts and relations used to represent the EDSS instrument. The instance EDSS-Pitié-Salpêtrière Hospital represents a specific EDSS instrument used at Pitié-Salpêtrière Hospital (Paris, France). The Figure illustrates the EDSSv1 for rating based on a Coded Score for neurological functions. In this case, the *NVA#59* hasForResult a Coded Score equal to “Minimal disability in two functional systems”. White rectangles represent domain ontology concepts; gray rectangles represent instances

Hence, the EDSS is both a Test-Instrument and a Neuroclinical Instrument that mainly measures disability in walking and motor control (see Table 2 for a full description). Figure 5 shows an excerpt of the classes and relations used to model the EDSS. The Subject Data Acquisition Instrument EDSS is a subclass of Neurologic Instrument. The domain explored by EDSS is Neurologic functions. An overall measurement of Disability Status is based on the EDSSv1 Variable (see Fig. 5), which combines the set of scores provided by the EDSS Sub-instruments.

**Table 2:** The neurologic instrument *Expanded Disability Status Scale (EDSS)* with its sub-instruments and instrument variables

Instrument model acronym	Variable model acronym	Domain explored by variable
Instrument model name		Quality measured by variable
EDSS	EDSSv1	Neurologic functions
Expanded Disability Status Scale		Disability status
EDSS-1	EDSS-1v1	Visual Function
EDSS Visual optic functions		Optic function performance
EDSS-2	EDSS-2v1	Cranial Nerves Function
EDSS Cranial nerve examination		Brainstem function performance
EDSS-3	EDSS-3v1	Motor Function
EDSS Pyramidal functions		Pyramidal function performance
EDSS-4	EDSS-4v1	Cerebellar Functions
EDSS Cerebellar examination		Cerebellar function performance
EDSS-5	EDSS-5v1	Sensory Function
EDSS Sensory examination		Sensory function performance
EDSS-6	EDSS-6v1	Bowel and Bladder Function
EDSS Bowel bladder functions		Bowel bladder function performance
EDSS-7	EDSS-7v1	Cerebral Functions
EDSS Mental status examination		Cerebral function performance
EDSS-8		Ambulation
EDSS Ambulation	EDSS-8v1	Ambulation performance without assistance
	EDSS-8v2	Ambulation performance with unilateral assistance
	EDSS-8v3	Ambulation performance with bilateral assistance

EDSSv1 is a subclass of Coded Variable and EDSSv1 hasForScale a Scale that hasForMinimumScaleItem: “0.0: Normal neurologic exam” and hasForMaximumScaleItem: “10.0: Death due to MS”. The whole scale is presented in Table 3. EDDSv1 isADataOf a Coded Variable Assessment and hasForResult a Coded Score, a subclass of both Score and Scale item. Most Scale items used in EDSS are Bi-coded Scale Items, i.e. Scale Items that have both a qualitative scale item code and a quantitative scale item code.

**Table 3:** The detail of the EDSSv1Variable and its linked Scale. The first and second left-most columns respectively contain the values taken by has quantitative scale item code and has qualitative scale item code properties

Min-value	Max-value	Number referred to by quantitative scale item	Value of quantitative scale item code	Value of qualitative scale item code
Yes	No	0.0	0.0	Normal neurologic exam (all grade 0 in all Functional System (FS) scores).
No	No	1.0	1.0	No disability, minimal signs in one FS (i.e., grade 1).
No	No	1.5	1.5	No disability, minimal signs in more than one FS (more than 1 FS grade 1).
No	No	2.0	2.0	Minimal disability in one FS.
No	No	2.5	2.5	Minimal disability in two FSs.
No	No	3.0	3.0	Moderate disability in one FS or mild disability in three or four FS though fully ambulatory.
No	No	3.5	3.5	Fully ambulatory but with moderate disability in one FS and one or two FSs grade 2.
No	No	4.0	4.0	Fully ambulatory without aid, self-sufficient, able to walk without aid or rest some 500 m.
No	No	4.5	4.5	Fully ambulatory without aid; able to walk without aid or rest some 300 m.
No	No	5.0	5.0	Ambulatory without aid or rest for about 200 m; disability severe enough to impair full daily activities.
No	No	5.5	5.5	Ambulatory without aid for about 100 m.
No	No	6.0	6.0	Intermittent or unilateral constant assistance required to walk about 100 m with or without resting.
No	No	6.5	6.5	Constant bilateral assistance required to walk about 20 m without resting.
No	No	7.0	7.0	Unable to walk beyond approximately 5 m even with aid, essentially restricted to wheelchair.
No	No	7.5	7.5	Unable to take more than a few steps; restricted to wheelchair; may need aid in transfer.
No	No	8.0	8.0	Essentially restricted to bed or chair or perambulated in wheelchair; generally has effective use of arms.
No	No	8.5	8.5	Essentially restricted to bed much of day; has some effective use of arm(s); retains some self-care functions.
No	No	9.0	9.0	Helpless bed patient; can communicate and eat.
No	No	9.5	9.5	Totally helpless bed patient; unable to communicate effectively or eat/swallow.
No	Yes	10.0	10.0	Death due to MS.

### The Clinical Dementia Rating as an Example of a Behavioral Instrument

**Fig. 6:**
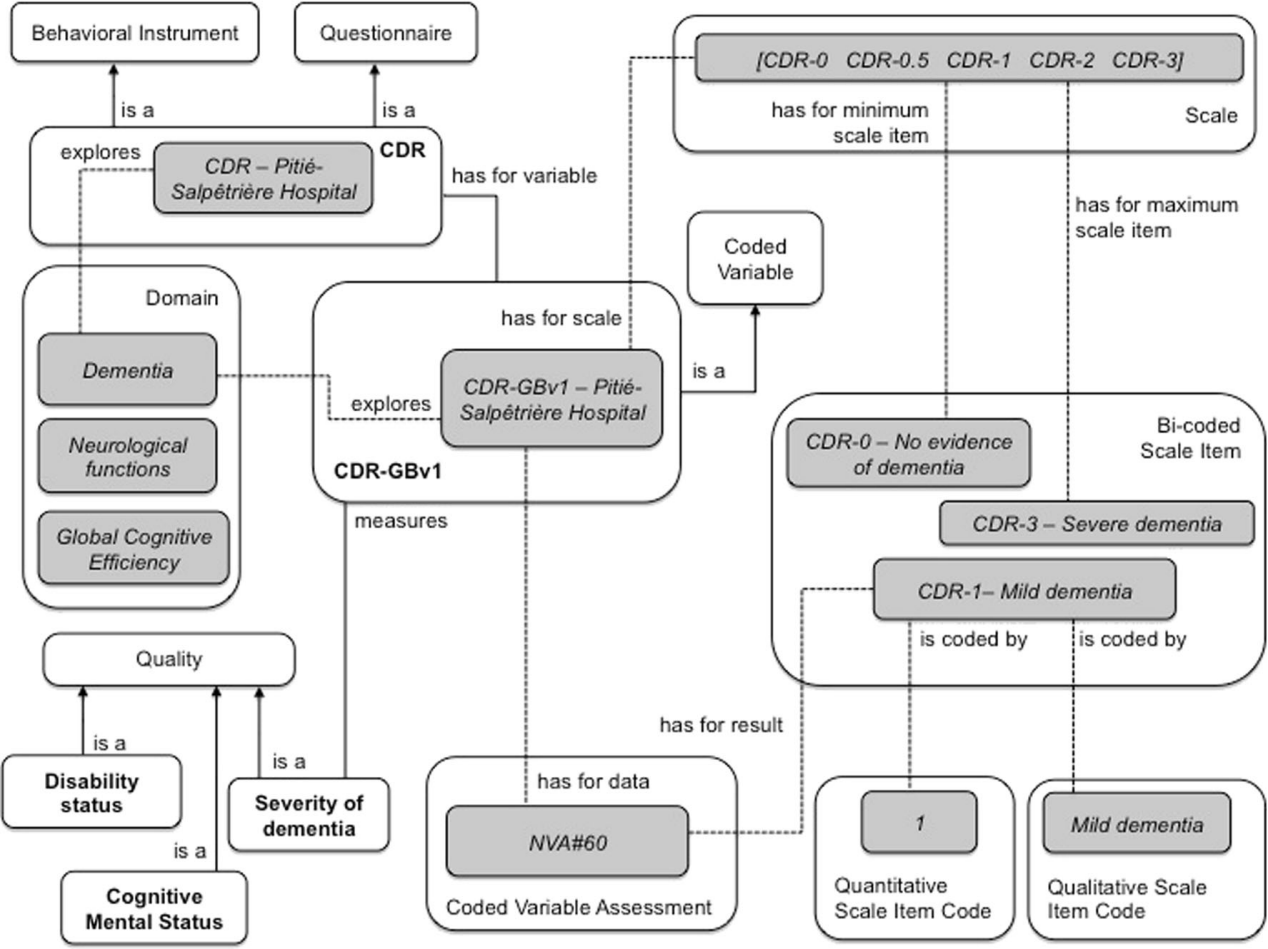
The main concepts and relations used to represent the CDR instrument. The variable CDR-SoBv1 is a subclass of Coded Variable and measures the quality Severity of dementia by combining scores from various sub-instruments. CDR-GBv1 hasForScale a Scale that hasForMinimumScaleItem “CDR-0: no evidence of dementia” and hasForMaximumScaleItem “CDR-3: severe dementia”. CDR-GBv1 isADataOf a Coded Variable Assessment that hasForResult a Coded Score - one of the Scale items that isAnAtomicPartOf Scale-CDR-GBv1 (between CDR-0 and CDR-3). These Scale Items are also Bi-coded Scale items

The Clinical Dementia Rating (CDR) was developed at the Memory and Aging Project at Washington University School of Medicine in 1979 for the evaluation of staging severity of dementia. The CDR is obtained through semi-structured interviews of patients (e.g. the clinician asks the question “Can you find your way around familiar streets? Usually, Sometimes, Rarely or Don’t Know”) and dementia is rated in 6 domains of functioning: memory, orientation, judgment and problem solving, community affairs, home and hobbies, and personal care; each of which is explored by a Sub-instrument (see Table 4) (Morris 1983). The CDR is modeled as a subclass of a Behavioral Instrument and a Questionnaire. The CDR is an illustrative example of an Instrument that investigates behavior, e.g. depression, anxiety or dependence (in dementia, for instance). These instruments are administered by Psychiatrists, Psychologists and (sometimes) Neurologists during a Behavioral Interview, which is a Questionnaire-based Assessment (see Fig. 2). Figure 6 shows an excerpt of the classes and relations used to model the CDR.

**Table 4:** The instrument Clinical Dementia Rating Scale *(CDR)* with its sub-instruments and instrument variables

Instrument model acronym	Variable model acronym	Domain expolred by variable
Instrument model name		Quality measured by variable
CDR	CDR-SoBv1	Dementia
		Severity of dementia (numerical Value [0,18])
CDR scale	CDR-GBv1	Dementia
		Severity of dementia (five-point scale)
CRD-M	CDR-Mv1	Memory
CDR-Memory		Severity of memory loss
CDR-O	CDR-Ov1	Orientation
CDR-Orientation		Severity of orientation difficulty
CDR-J	CDR-Jv1	Problem solving judgment
CDR-Judgment and problem solving		Severity of impairment in solving problems
CDR-CA	CDR-CAv1	Community-activities
CDR-Community affairs		Severity of impairment in community activities
CDR-HH	CDR-HHv1	Home-activities
CDR-Home activities and hobbies		Severity of impairment in home activities
CDR-PC	CDR-PCv1	Personal care
CDP-personal care		Level of dependency

Each domain is rated on a 5-point scale of functioning as follows: 0, no impairment; 0.5, questionable impairment; 1, mild impairment; 2, moderate impairment; and 3, severe impairment (personal care is scored on a 4-point scale without a 0.5 rating available). The global CDR score is computed using the Washington University online algorithm.^13^ The domain Dementia is rated with a global measurement (CDR-SoBv1 variable) obtained by summing each of the domain box scores, with scores ranging from 0 to 18. The Numerical Variable (CDR-SoBv1) is transformed into a Coded Variable(CDR-GBBv1) which hasforscale a 5-point scale in which CDR-0 connotes no cognitive impairment, and then the remaining four points are for various stages of dementia: CDR-0.5 = very mild dementia (questionable dementia), CDR-1 = mild, CDR-2 = moderate, CDR-3 = severe (see Table 5).

**Table 5:** Details of the CDR-GBv1 Variable and its related scale Scale-CDR-SoBv1

Min-value	Max-value	Number referred to by quantitative scale item	Value of quantitative scale item code	Value of qualitative scale item code
Yes	no	0	CDR-0	No evidence of dementia
No	no	0.5	CDR-0.5	Questionable dementia
No	no	1	CDR-1	Mild dementia
No	no	2	CDR-2	Moderate dementia
No	yes	3	CDR-3	Severe dementia

The instance CDR-Pitié-Salpêtrière Hospital represents a specific CDR instrument used at Pitié-Salpêtrière Hospital (Paris, France). In this case, the *NVA#60* hasForResult a Coded Score equal to “mild dementia”.

A white rectangle represents a domain ontology concept; a gray one represents an instance.

## Discussion

We shall first discuss the methodology used to build the ontology and then position our work within the field of ontological engineering in general and the engineering of biomedical ontologies in particular (§5.1). Lastly, we shall focus on our results, the ontology of instruments used in neurosciences, its current content, future extensions and connections with services (reasoning) supported by the model (§5.2).

### Engineering Biomedical Ontologies

The “multi-abstraction-layer” and “multicomponent” approach adopted here is generally advocated for the design of large, multi-domain ontologies (Borgo and Masolo 2009) in general and those used in biomedical research in particular (Smith and Scheuermann 2011).

In theory, this type of approach has several advantages. Firstly, the use of foundational ontologies impacts the overall quality and consistency of domain ontologies. Secondly, the development of core domain ontologies is more likely to produce inter-domain consistency and avoids the proliferation of concepts and relations. Indeed, our experience has revealed the following positive aspects. The joint use of DOLCE, IEC (Fortier and Kassel 2004) and a generic ontology of artifacts (Kassel 2010) allowed us to distinguish between three complementary dimensions for our assessment instruments. The intrinsic properties of instruments correspond to conceptual contents that are physically materialized by documents. The instruments’ functional properties are qualities of entities corresponding to the domains that they explore, whereas the instruments’ social properties are created and maintained by communities of practice. Moreover, a core ontology of these instruments leads to homogeneous conceptualization, which impacts on both the exploitation of the derived domain ontologies (i.e. uniformity of queries) and the ontologies’ maintenance.

This multilayer approach uses a foundational ontology to structure the conceptualization. The question of which foundational ontology to choose then arises. In the biomedical domain, the Open Biological and Biomedical Ontologies (OBO) initiative has federated a large group of researchers around the Basic Formal Ontology (BFO) and the reference ontology of relations (Smith et al. 2007). Other initiatives are exploring alternatives to BFO. As we have seen, DOLCE has been used for the development of OntoNeuroLOG. In fact, the two foundational ontologies are quite similar. Our choice of DOLCE was dictated by two main factors. On one hand, its cognitive bias proved to be suitable for the modeling of assessment instruments having a cognitive (social) nature (in contrast to BFO’s realistic stance). On the other hand, the availability of a complete, rigorous axiomatic (i.e. a set of axioms) facilitated the understanding (and therefore the reuse) of the foundational ontology (Temal et al. 2008). However, over and above the choice of one foundational ontology or another, what is really important is the existence of modules that complement the foundational ontology and the modules’ overall structure (Schneider et al. 2011). We used a generic ontology of artifacts and a general concept of artifact that transcends the domains of physical entities and mental and social entities. This represents an important element of our model because it enables the conceptualization of instruments as subclasses of artifacts. In the OBI (Brinkman et al. 2010) extension of the BFO, instruments are considered only as material, physical entities (the term “device” is used synonymous with “instrument”). Questionnaires are also introduced. However, their artifact status, intentional origin and function are all ignored. This observation shows the importance of defining a more generic concept of “measuring instrument” (artifact, in our case) and anchoring the latter within a foundational ontology.

We were confronted with two main limitations of DOLCE - limitations that also affect other foundational ontologies, as far as we know. The first limitation concerns the conceptualization of the values of Qualities (Qualia) and regions of values (Quality Spaces) as abstract entities with no spatiotemporal location (Abstracts). In keeping with the notion of a *region of values*, we considered measurement scales to be Quality Spaces. However, as noted in Section 3, measurement scales are created, adopted and removed. Indeed, they have the same temporal extension as the assessment instruments to which they are attached. This clearly contradicts the timeless nature of Abstract entities. Secondly, different instruments with different measurement scales can measure the same kind of Quality; hence, our current association between a single Quality Space and one kind of Quality is not tenable. These two limitations have been acknowledged by the authors of DOLCE and removed from the revised DOLCE-CORE kernel (Borgo and Masolo 2009). Fundamentally, a new view of the nature of the Qualities was introduced by making them depend on standardized measuring instruments (Masolo 2010); what we measure depends not only on the measurement procedure but also (and above all) on the instruments created for this purpose. In parallel with this revision, two extensions were recently proposed: an ontology of semantic data^14^ (Probst 2008) and a generic ontology of observation and measurement (Kuhn 2009). These works^15^ demonstrate the vitality of research in the field of foundational ontologies and emphasize the need for maintenance of ontologies (such as the one presented here) so that they can take account of on-going developments and newly identified needs.

Conceptually, formalization facilitates future ontology extensions, ontology reuse and inter-ontology interoperability (such DOLCE and BFO). However, only dissemination into several domain ontology applications (such as those proposed in this paper) can provide us with feedback on the strengths and weaknesses of our approach.

### Which Instrument Models Should Be Used for Which Purposes?

As we saw in Section 4, OntoNeuroLOG covers a wide range of instruments - from tests to questionnaires. Along with the ontology’s scope in terms of domains covered, another important aspect relates to its functional scope (i.e. the kinds of reasoning that it enables). The latter is related to the point of view adopted and the level of detail considered when modeling the instruments.

Since our current objective is to share scores within a federation of research centers, we combined a structural description of instruments with a representation of the words used to name the variables and items of scales. Scores are shared through the definition of standard instruments to which locally administered instruments conform to a varying degree. In this respect, it is important to note that today’s ontology representation languages fail to account correctly for our knowledge of instruments. As we have seen, OntoNeuroLOG’s classes represent standard instruments with a standard terminology, whereas a local instrument is conceptualized as an instance of one of these classes. This forces the descriptions of the local instrument and acquired scores to be logically consistent with the description of a class of standard instrument. As a consequence, it is not possible to represent variations of a local instrument with respect to a standard instrument (e.g. a local questionnaire that has one or more items that the standard instrument or that measures a variable with a different scale). Indeed, Hoehndorf et al. have shown that it is impossible to correctly represent default knowledge in classes (Hoehndorf et al. 2007). Hence, our descriptions of local instruments are made logically consistent with the descriptions of standard instruments. We do not formalize variations and thus leave them in the documentation.

Our ontology reflects the functional dimension of instruments to some extent: instruments “explore” domains and variables “measure” qualities. OntoNeuroLOG is primarily based on the clinical expertise acquired at Pitié-Salpêtrière Hospital. Although domains are modeled here as concepts (i.e. instances of a Concept class) with which a term is associated (e.g. “long term verbal memory” or “problem solving and judgment”), the classes of entities to which these terms refer are generally not conceptualized in the literature. Here, we faced two challenges: firstly, as underlined by (Bilder et al. 2009), the terms used to describe the domain of instruments and the measured qualities are not sufficiently well defined: for example, does the term “memory” refer to a capacity, a function or a process? Does the term “working memory” mean a kind of “memory”? These terms are vague and there is no consensus on their definition (Bilder et al. 2009). For example, what exactly are the differences between “short-term memory” and “working memory” or between “episodic memory” and “declarative memory”? Secondly, several concepts in the field of foundational ontologies are subject to debate; a consensus on the notions of *process* (Galton and Mizoguchi 2009), *capacity*/*disposition* and *function* (Borgo and Masolo 2009) has not yet emerged.

Furthermore, there are two main issues related to detailed modeling of the instruments’ functional dimensions. Firstly, there is a need to improve the management of functional dimensions, by (for example) finding instruments that explore a given function or by comparing instrument definitions that change over time (White and Hauan 2002). Secondly, specifying the semantics of a given score (such as a measure of a given quality of a given entity) must yield a more accurate, detailed model of the subject and his/her state.

Again, these aspects show that our instrument ontology is not set in stone and will evolve, with (for example) the development of “cognitive” ontologies (Bilder et al. 2009). In this respect, one can note the recent work on Cognitive Paradigm Ontology (CogPO) (Turner and Laird 2012), which seeks to represent the experimental conditions (i.e. the types of stimuli, sequences, instructions and expected responses) used in fMRI and PET experiments. The CogPO is based on the Cognitive Atlas, a growing knowledge base that lists and classifies the concepts used in cognitive science (http://www.cognitiveatlas.org/).

One can also note the recent work on standardization undertaken by the US National Institute of Neurological Disorders and Stroke; the Common Data Elements (http://www.commondataelements.ninds.nih.gov/) initiative is aimed at harmonizing information provided in the context of Parkinson’s disease.

## Conclusion

Many neuroscience research centers and networks are now collecting neurologic, neuropsychological, behavioral and imaging data in large databases. In this context, we consider that our ontology of instruments is relevant for two main reasons.

Firstly, it represents an ontological repository based on in-depth clinical expertise acquired at Pitié-Salpêtrière Hospital - an institution known for its outstanding experience in neurology and neuropsychology. The exhaustive list of entities (instruments, variables, explored domains and measured qualities) reflects a broad, well-understood, clinical state of the art. Moreover, the output of this acquired expertise (in the form of documents describing instruments and variables) matched the clinical procedures applied in three other French university medical centers (in Rennes, Grenoble and Nice) that participated in the NeuroLOG project. The fact that about 70 % of the tests incorporated into OntoNeuroLOG were used in all four institutions justifies our on-going efforts to standardize neuropsychological and behavioral assessments.

Secondly, our ontology may be extended to the representation of imaging data and ancillary, behavioral, neuropsychological and cognitive data. It provides a coherent semantic space and a knowledge repository for structuring and designing a new generation of databases and associated services in neurosciences. OntoNeuroLOG may facilitate automatic reasoning and knowledge extraction from appropriately designed and structured databases. An example of a query that can be expressed using our structured ontological framework is the selection of instruments that measure variables related to memory dysfunction, the retrieval of subjects with imaging markers such as *cortical thickness* and neuropsychological markers such as *memory impairment* or *severe dementia,* and the retrieval of the associated scores from a battery of tests.

Our ontology’s target application is the management of large data repositories in neurology and psychiatry – fields of medicine that are being completely transformed by the recent introduction of multimodal imaging. We consider that the scheme class/instance proposed here offers ontology builders the flexibility needed to seamlessly introduce notions that are currently difficult to formalize (e.g. cognitive domains, such as verbal comprehension). We hypothesize that the development of the brain function ontology backed by some researchers (Bilder et al. 2009; Price and Friston 2005) will help to formalize cognitive notions and then introduce new classes (rather than instances) into our ontology.

## Information Sharing Statement

The ontology (ONL-MSA, Mental State Assessment (RRID:nlx_157474)) is available through the NCBO BioPortal ontologies (http://bioportal.bioontology.org/ontologies/ONL-MSA). OWL and Ontospec (a semi-informal ontology language) files can be downloaded from http://neurolog.unice.fr/ontoneurolog/v3.0/. Information about NeuroLOG project (ANR-06-TLOG-024) can be found on the wiki site at neurolog.i3s.unice.fr/.

## Footnotes

1 The NeuroLOG project (2007–2010) was funded by the French National Agency for Research. http://neurolog.i3s.unice.fr/neurolog/

2
http://www.birncommunity.org/

3 According to Pubmed, MMS, EDSS and CDR were respectively cited in 3043, 872 and 1070 publications over the period 2008–2012.

4 With respect to our notation, the informal labels on DOLCE’s categories appear in the text in Courier New font with First Capital Letters for the concepts and a javaLikeNotation for relations. The same conventions apply to all the ontologies presented in the present paper.

5 Due to space limitations, we only provide brief descriptions of DOLCE’s categories and those used in our ontology of instruments. For a complete presentation, the reader is invited to refer to (Trypuz 2008).

6 The term “semiotics” is used here with reference to the roles of *signifier* and *signified* played at different times by the entities Inscription, Expression and Conceptualization. Thus, when we say that an Expression accounts for a Conceptualization, we consider that the Conceptualization is the result of an interpretation by an agent, with the Expression playing the role of *signifier* and the Conceptualization being the *signified*.

7 Because these constraints depend on local usages and/or national legislations, the core ontology does not provide with a set of predefined constraints. Constraints can be specified using the ‘isEmpoweredToPerform’ relationship in the module Action-OS.

8 CDISC (Clinical Data Interchange Standards Consortium), CDISC Clinical Research Glossary Version 7.0, Applied Clinical Trials (2008), 12–58. (see http://www.cdisc.org/glossary/CDISC2008GlossaryVersion7.0.pdf).

9 These documents correspond to what are commonly called “test materials” (American Psychological Association, 2002), i.e. protocols, manuals, test items, scoring algorithms and so on. Technically, we identify an Instrument as propositional content reifying the contents of all the documents that materialize an instrument.

10 Even apparently basic tasks may be “complex”. The current connectionist view is that the brain operates via a number of “distributed functions and subsystems”.

11
*Reification*: The term ‘reification’ commonly means ‘making something concrete’. In our case, concepts that refer to classes of entities are made concrete by considering them as individuals modeled by instances.

12 At a same time, billions of people on the planet have the same corporal temperature. More precisely, according to the theory of Qualities adopted in DOLCE, it corresponds to the population of the Earth for billions of Temperature Qualities (one per person), but many of them share the same value (Quale) at a given time.

13
http://www.biostat.wustl.edu/~adrc/cdrpgm/index.html

14 In the philosophy of mind, “sensory data” (“qualia”) are distinguished from “semantic data” to which linguistic terms are related and which are used to communicate observations and measurement results (for a comprehensive overview of this distinction, see http://en.wikipedia.org/wiki/Qualia).

15 The OASIS consortium has defined a standard ontology for quantities and units of measure (http://www.oasis-open.org/committees/tc_home.php?wg_abbrev=quomos).
